# Melatonin Receptors and Serotonin: Age-Related Changes in the Ovaries

**DOI:** 10.3390/jpm14091009

**Published:** 2024-09-22

**Authors:** Victoria Polyakova, Dmitrii Medvedev, Natalia Linkova, Mikhail Mushkin, Alexander Muraviev, Alexander Krasichkov, Anastasiia Dyatlova, Yanina Ivanova, Giuseppe Gullo, Anna Andreevna Gorelova

**Affiliations:** 1 St. Petersburg Research Institute of Phthisiopulmonology, 2-4 Ligovskii Ave., 191036 St. Petersburg, Russiaivanova.yana803@gmail.com (Y.I.); 2 The Laboratory “Problems of Aging,” Belgorod National Research University, 308015 Belgorod, Russia; 3 St. Petersburg Institute of Bioregulation and Gerontology, 3 Dynamo Ave., 197110 St. Petersburg, Russia; 4 The Department of Social Rehabilitation and Occupational Therapy, St. Petersburg Medical and Social Institute, 72A Kondratievsky St., 195271 St. Petersburg, Russia; 5 Department of Traumatology and Orthopedics, Pavlov First St. Petersburg State Medical University, L’va Tolstogo str., 6-8, 197022 St. Petersburg, Russia; mikhail_mushkin@mail.ru; 6 Department of Radio Engineering Systems of Electrotechnical University LETI, 5F Prof. Popova Str., 197022 St. Petersburg, Russia; 7 Obstetrics and Gynecology Unit, Villa Sofia Cervello Hospital, I.V.F. Public Center, University of Palermo, Via Trabucco, 180, 90146 Palermo, Italy; gullogiuseppe@libero.it; 8 Department of Urology, Medical Institute, St. Petersburg University, Mendeleevskaya Line 2, 197036 St. Petersburg, Russia; gorelovauro@gmail.com

**Keywords:** melatonin receptors, *MTNR1A* and *MTNR1B* genes, serotonin, ovary, ontogenesis, aging

## Abstract

Introduction. Melatonin and serotonin can influence certain aging processes in the ovaries. The main melatonin receptors are represented by types MT1 and MT2. The goal of investigation. Here, we evaluated the expression of genes and synthesis of MT1 and MT2 receptors, as well as serotonin synthesis in the ovaries during ontogenesis. Methods. We analyzed histological material obtained from the ovaries of infants, women of younger and older reproductive age, premenopausal, menopausal, and postmenopausal women. For the analysis of MT1 and MT2 receptors and serotonin expression and synthesis, RT-PCR and immunohistochemistry were used. Results. We found that the synthesis of serotonin, as well as MT1 and MT2 receptors in the ovaries significantly decrease in ontogenesis. The sharpest drop in these molecules was observed in samples obtained from one-year-old infants, as well as from pubescent girls and menopausal women. A statistically significant 2.3–7.6-fold decrease in the expression of *MTNR1A* and *MTNR1B* genes in the ovaries was also observed in one-year-old infants, in adolescents, and in middle-aged women. Conclusions. These data are crucial to understanding the fundamental mechanisms of aging of the female reproductive system and the search for molecules predicting its aging.

## 1. Introduction

Melatonin (MT) is a hormone secreted and synthesized by the pineal gland. The melatonin circadian cycle contributes to the regulation of body temperature, the secretion of reproductive hormones, e.g., luteinizing hormone, and the circadian sleep/wake cycle. It was thought that melatonin is formed exclusively in the pineal gland; however, it has been established that this molecule is synthesized beyond the epiphysis. The extrapineal production of melatonin was discovered by I.M. Kvetnoy and N.T. Raikhlin in 1975, when they established that the synthesis of melatonin can occur in the cells of the appendix [1]. Further, it was revealed that melatonin synthesis occurs in other parts of the neuro-immunoendocrine system, such as the retina, cerebellum, mucous membrane of the respiratory tract, liver, kidneys, adrenal glands, thymus, bone marrow, lymphocytes, platelets, etc. [2,3,4].

Local melatonin synthesis also takes place in the cells of the female reproductive system [5]. This was first suggested by the detection of melatonin and its precursors, serotonin and N-acetylserotonin, and the activity of AANAT (Aralkylamine N-acetyltransferase) and HIOMT (Hydroxy-Indole-O-Methyl Transferase) in human ovarian extracts [6]. Interestingly, the melatonin concentration found in human ovarian follicular fluid is almost triple the concentration of that found in simultaneously-drawn blood samples [7]. The melatonin level in follicular fluid is probably determined by several factors, such as the accumulation of melatonin from the blood and local melatonin synthesis by granulose follicle cells, granulose oocyte cells, mast cells [8,9]. Melatonin from ovarian cells cannot be passed into the general bloodstream. Ovarian cells use the produced melatonin for their own or adjacent cells’ needs; that is, as an antioxidant, autocrine, or paracrine agent [10].

Melatonin contributes to oocyte maturation, fertilization, and the development of embryos [11]. Dysregulation of these processes can cause infertility. For example, luteinized unruptured follicle syndrome can develop against the backdrop of endocrine and immune system dysfunction. It can be associated with melatonin synthesis problems [12]. Moreover, melatonin regulates thyroid gland function. Thyroid autoimmunity is considered to be one of the main causes of female infertility. These data emphasize the leading role of melatonin for the female reproductive system’s function [13]. Melatonin, in combination with other biologically-active substances, can normalize the functioning of a woman’s reproductive system [14]. Moreover, melatonin reduces the oxidative damage to oocytes incubated with oxidants and improves IVF results [15].

The effects of melatonin are mediated by binding to its receptors. Receptors of melatonin (MT) are divided into membrane and nuclear receptors, respectively. The nuclear receptor is related to the RZR/ROR nuclear receptor superfamily [16].

In 1999, the expression of melatonin membrane receptors (MT1, MT2) was identified in oocytes and granulosa cells [17]. The MT1 receptor is widespread in the ovaries. The MT1 receptor is crucial for melatonin-mediated protection against ovarian damage caused by cisplatin, a chemotherapeutic agent which inhibits cellular mitosis [18].

Here, we provide our complex analysis of melatonin synthesis in ovarian tissue during ontogenesis, based on the synthesis of MT1 and MT2 receptors, as well as the melatonin precursor serotonin. The aim of the study is the comparative expression assessment of melatonin receptors and serotonin in ovarian tissue of women of different ages.

## 2. Materials and Methods

### 2.1. Characteristics of Experimental Groups

The study was performed on autopsy material from female patients (n = 164). The material was obtained from archived autopsy specimens of ovaries. The groups were divided as follows:

Group 1: infants under 12 months (antenatal) (n = 10);
Group 2: 2–4-year-old infants (n = 5),
Group 3: 5–11-year-old girls (n = 5),
Group 4: 12–15-year-old girls (n = 11),
Group 5: women of the younger reproductive ages of 16–18 (n = 20),
Group 6: women of the reproductive ages of 19–29 (n = 23),
Group 7: women of the older reproductive ages of 30–44 (n = 24),
Group 8: premenopausal and menopausal women of ages 45–59 (n = 21),
Group 9: menopausal women of ages 60–74 (n = 19),
Group 9: postmenopausal women of ages 60–74 (n = 10),
Group 10: postmenopausal women of ages 75–89 (n = 10),
Group 11: postmenopausal women of ages 90–92 (n = 6).

The criteria for exclusion from the study were pathologies of reproductive and endocrine systems for the control group. All female patients included in the study had no pathologies of the endocrine system and had not used hormonal or any other medications for 3 years prior to the study.

The expression of *MTNR1A* and *MTNR1B* genes in the ovaries was determined by PCR and compared in different age groups. Synthesis of MT1 and MT2 melatonin receptors, as well as serotonin, were assessed by the immunofluorescence method and their quality was assessed by morphometric analysis. The study design was approved by the ethics committee at the St. Petersburg State Research Institute of Phthisiopulmonology (Project identification code—N3/22; date: 15 June 2022). The morpho-functional state of the ovaries was analyzed using clinical data and the conclusions of pathomorphological examinations conducted by the Department of Anatomical Pathology at St. Petersburg State Pediatric Medical University and the Department of Morbid Anatomy at St. George Hospital.

### 2.2. Immunofluorescence Staining

The paraffin sections (4–6 μm thick) were mounted on microscope slides with poly-L-lysine coating (Sigma, Fukushima, Japan). The study utilized the immunocytochemical method using primary monoclonal antibodies to MT1 (1:100, Abcam, Cambridge, UK) and MT2 (1:150, Abcam). Antibodies conjugated to Alexa Fluor 647 fluorochrome (1:1000, Abcam) were used as secondary antibodies for the immunofluorescence reaction. Sections were incubated for 30 min at room temperature in the dark. Cell nuclei were visualized by Hoechst 33,258 (Sigma, Livonia, MI, USA).

As a negative control, the immunohistochemical reaction was performed without using primary antibodies. Antibody specificity was verified in control experiments.

### 2.3. Morphometrical Analysis

The immunofluorescence staining results were visualized using a Zeiss LSM 980 confocal microscope (Zeiss, Oberkochen, Germany). Preparations were stained using a standard protocol. Morphometry was performed with Image J (1.8.0_112) software to assess the relative area of expression, which was calculated as a ratio of the immunopositive reaction area to the total tissue area in the field of view and expressed as a percentage. For each specimen, five microphotographs were obtained at ×200 magnification. Expression area (%) was calculated as the ratio of the area occupied by immunopositive cells to the total field of view.

### 2.4. Statistical Processing

To determine the statistical significance of differences between the quantitative parameters of normally distributed data from the groups under study, analysis of variance (ANOVA) and dispersion and the Kolmogorov–Smirnov criterion were used. The abnormal nature of the distribution of quantitative data has been established. Given the abnormal nature of the distribution, when describing the results, the data are presented as a median (Q25; Q75). In the case of nonparametric data distribution, we utilized the Kruskal–Wallis test, median test, and Dunn’s test. The hypothesis of the equality of mean values in the groups under study was discarded at *p* (probability value) < 0.05. Student’s *t*-test was used to evaluate intergroup differences in the case of normally distributed data. Statistical analysis was performed using Statistica 7.0 software.

### 2.5. PCR

Total RNA from the ovary tissue was stabilized using an IntactRNA RNA stabilization solution (Evrogen, Moscow, Russia). RNA isolation was performed using an RNeasy MiniKit (Qiagen, Hilden, Germany) in accordance with the manufacturer’s recommendations. Spectrophotometric analysis was used to assess RNA purity. The ratio of optical density at wavelengths of 260 and 280 nm (A 260/280) was about 2. The integrity of each RNA sample was estimated based on fragment size distribution indicated by two peaks corresponding to 18S and 28S ribosomal RNAs and a signal from small RNAs. The quality of RNA was assessed based on RNA integrity number (RIN) values ranging from 1 to 10, with 1 being the most degraded and 10 being the most intact, using an RIN algorithm [19]. The RNA integrity number (RIN) in our investigation was 8 [20]. The concentration range of isolated RNA was near 800–1000 ng/μL. The first strand of cDNA was synthesized via a Revert Aid First Strand cDNA Synthesis Kit (Thermo Fisher Scientific Inc., Waltham, MA, USA) using 100 ng of RNA per 20 µL of the reaction mixture. The obtained cDNA was used directly as a template for quantitative PCR at 1 µL per 24 µL of the reaction mixture. Quantitative PCR was performed by means of a DT-322 (DNK-Technology, Moscow, Russia) using a qPCRmix-HS SYBR + ROX amplification kit (Evrogen, Russia). Quantitative PCR was used to quantify the expression of MTNR1A and MTNR1B genes. Oligonucleotide primers were designed using the NCBI Primer-Blast online service. Primer pairs, in which one of the primers corresponded to regions of two adjacent exons, were used. The synthesis of oligonucleotides was carried out at NPO Syntol (Moscow, Russia). The primer sequences for *MTNR1A* and *MTNR1B* genes are presented in Table 1. The expression level relative to the reference GAPDH housekeeping gene was determined by the 2^−ΔΔCq^ method. Statistical processing of the results and plotting of the diagrams was carried out via Microsoft Excel 2021. Three independent samples from each group were investigated. A minimum of three parallel reactions in adjacent slots were performed.

## 3. Results

### 3.1. MT1, MT2, and Serotonin Expression in Human Ovarian Tissue from Different Age Groups

#### 3.1.1. Results of the Immunofluorescence Analysis of Ovarian Tissue

To determine MT1 and MT2, as well as serotonin levels in ovarian tissue, the immunofluorescence method was used. Table 1 and Table 2 present the results of a quantitative analysis of expression area and optical brightness, respectively.

The expression area of the signal molecules in the ovaries tended to decrease from the antenatal period (under 12 months) to 19–29 years: by 13.3-fold for MT1 and 18.1-fold for MT2. Thereafter, the decrease proceeded less intensively, reflecting a plateau of the studied parameters and reaching median values of 0.76% for MT1 and 0.25% for MT2 in groups over 90 years of age.

**Figure 1:**
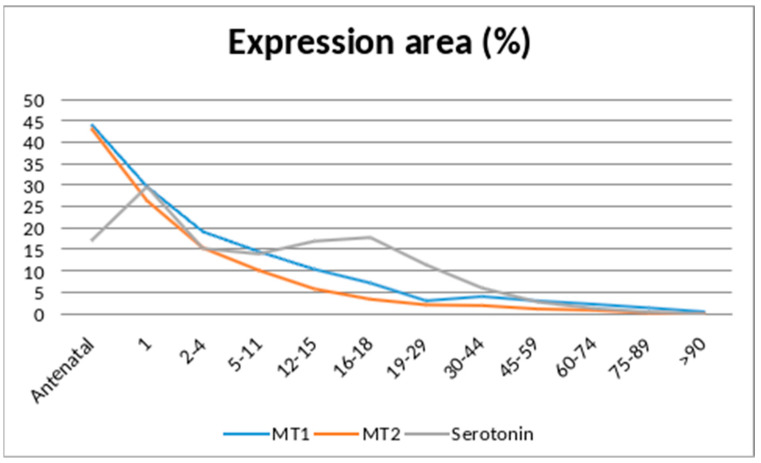
Dynamics of the expression area of MT1, MT2, and serotonin in ovarian tissue of women in different age groups.

The expression of the serotonin-signaling molecule in the ovarian tissue tended to be twice that of the antenatal period, with its subsequent halving by 5–11 years. After a slight elevation in the size of the expression area by the age of 16–18, its gradual decrease was noted without the formation of peaks in the rise of the indicator (Figure 1).

The optical brightness of MT1 and MT2 receptors in the ovaries tended to decrease systematically from the antenatal period without the formation of any increases in the studied age groups. For serotonin, an increase in the optical brightness of receptors was detected with the formation of two peak rises in values from 1 y.o. to 2–4 y.o. (1.5-fold) and from 5–11 y.o. to 16–18 y.o. (2-fold). Further, a systematic decrease in the optical brightness of serotonin receptors was determined without the formation of areas of elevation of the studied parameter. The results of measurements of optical brightness are presented in Table 3.

**Figure 2:**
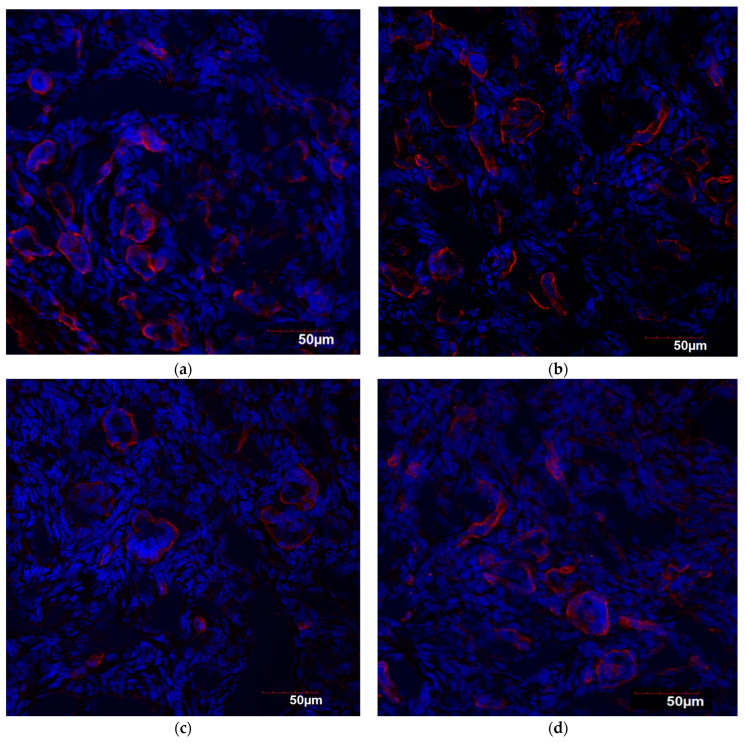

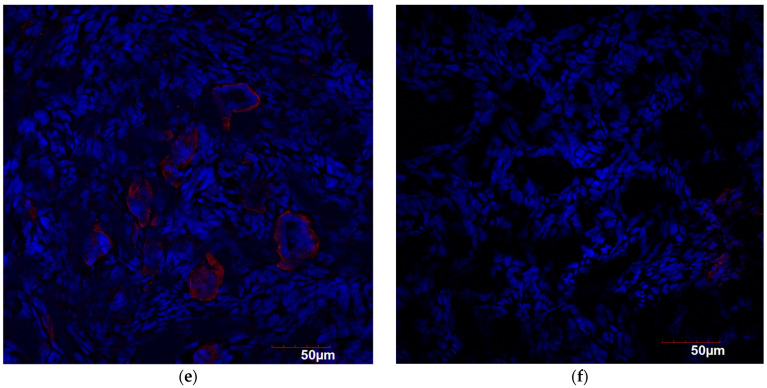
MT1 and MT2 expression (red staining) in human ovarian tissue of women in different age groups. Confocal microscopy (cell nuclei stained with Hoechst), magnification 200: (**a**) *MT1* and (**b**) *MT2*—children (1 y.o.); (**c**) *MT1* and (**d**) *MT2*—19–29 y.o.; (**e**) *MT1* and (**f**) *MT2*—60–74 y.o.

Microphotographs with results of the immunofluorescence staining of the studied signal molecules are presented in Figure 2.

#### 3.1.2. Results of MTNR1A and MTNR1B PCR Analysis of Ovarian Tissue

**Figure 3:**
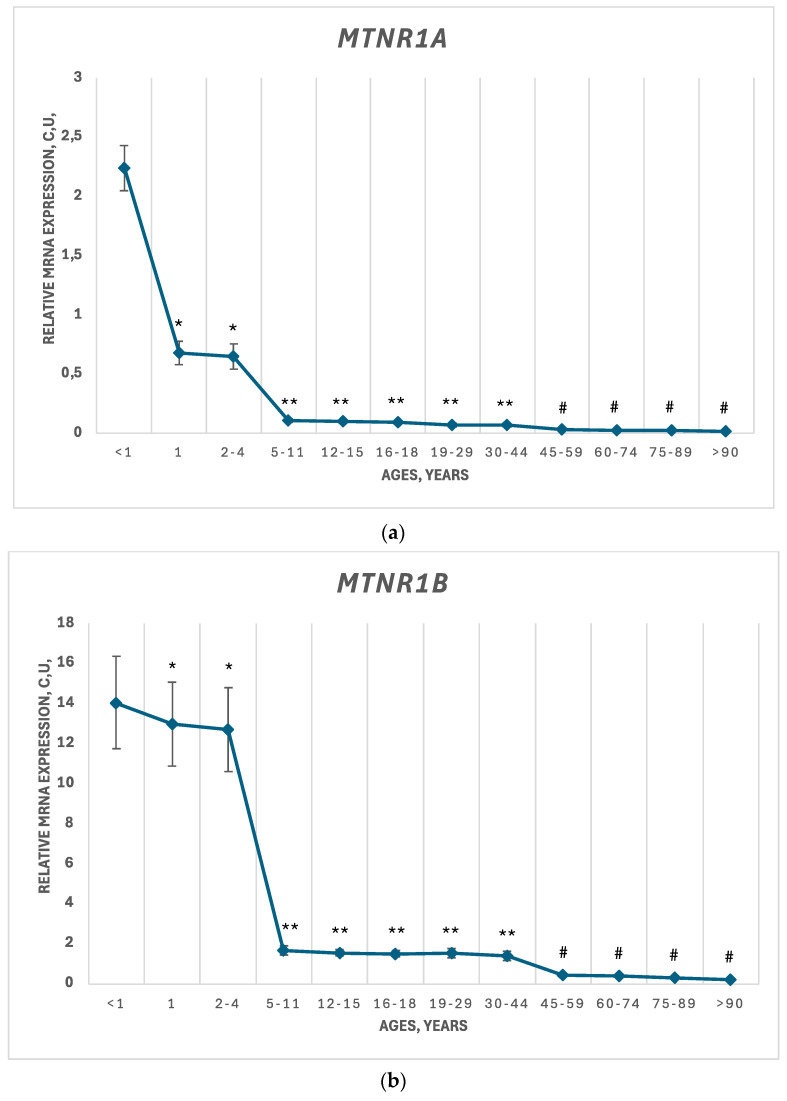
Relative mRNA expression (c.u.) of *MTNR1A* and *MTNR1B* genes and serotonin in ovarian tissue of women in different age groups. (**a**) *MT1—MTNR1A*; (**b**) *MT2—MTNR1B.* Data are presented as a mean and standard deviation. * *p* < 0.05 compared to the “Antenatal (<1)” group; ** *p* < 0.05 compared to the “1 y.o.” group; # *p* < 0.05 compared to the “19–29 y.o.” group.

For a deeper understanding of MT1 and MT2 expression in human ovarian tissue at different ages, a PCR-RT analysis of *MTNR1A* and *MTNR1B* genes was performed. The full data corresponding with previous results were estimated. For both genes, a decrease in mRNA expression over the course of a woman’s lifetime was shown. For the *MTNR1A* gene, which encodes MT1, a sharp drop was found during transition from the antenatal period to 1 y.o.; *MTNR1A* expression decreased by 3.3 times. The next period of dramatic decrease was observed in the transition from 2–4 y.o. to 5–11 y.o. (by almost 6 times). Thereafter, the decline was more subtle up to 90 y.o. It is worth noting that *MTNR1B* expression was dramatically higher than *MTNR1A* expression in all of the studied groups. In the antenatal group, this exaggeration was equal to 7, while in the 90 y.o. group it was almost 13 times. Although *MTNR1B* expression did not drop precipitously in transition from the antenatal period to 1 y.o., *MTNR1B* expression decreased 12-fold in transition from 2–4 y.o. to 5–11 y.o. Further decline was slighter up to 90 y.o. The results of the measurements are presented in Figure 3.

## 4. Discussion

It is thought that the level of melatonin in follicular fluid is a marker of ovarian reserve. It was revealed that intra-follicular melatonin level decreases with age, possibly due to the dysfunction of the granulosa cells and the pineal gland. The study encompassed a total of 61 women (23–45 years old) who were divided into three groups: poor, normal, and high ovarian response depending on their response to stimulation. Interestingly, the melatonin level in the follicular fluid on the day of oocyte extraction was the lowest in the group with poor ovarian response. This turned out to be 5 times less than in the group with an average response and 11 times less than in the group with a high response. When comparing IVF results among the groups, it was noted that patients in the group with a high ovarian response, who also had the highest level of melatonin in their follicular fluid, had the best IVF results. Melatonin levels significantly correlate with the parameters of IVF results [21].

It was shown that the levels of melatonin and extracellular DNA in intrafollicular fluid are closely associated with IVF outcomes. The study included a total of 325 patients who were divided into two groups after the IVF procedure: pregnant and not pregnant. The average age was 30. The melatonin level in the follicular fluid of the first group was higher than that of the second group by a factor of 1.4. Intra-follicular melatonin concentration had a significant impact on the fragmentation rate and the proportion of high-quality embryos on day three. It was noted that the melatonin level was significantly higher in the follicular fluid samples corresponding to high-quality embryos in pregnant women [22].

It is believed that the above effects of melatonin are carried out through binding to its receptors. It has been shown that melatonin receptors are partially responsible for melatonin-induced positive effects on embryonic development. Melatonin can suppress the expression of pro-apoptotic genes, including Bax and Caspase-3, and activate the expression of the anti-apoptotic Bcl-2 gene, thereby reducing the rate of blastocyst apoptosis and improving the quality of embryos and the subsequent rate of embryo implantation. Experiments on switching off receptors have shown that MT1 and MT2 demonstrate an anti-apoptotic effect [23].

Melatonin is also able to influence certain aging processes in the ovaries and oocytes via various other mechanisms. Ovarian aging is characterized by a gradual decrease in the number and quality of oocytes, which can lead to infertility. Although the molecular mechanism of the reduction in the quantity and quality of oocytes is not fully understood, the aging process of the ovaries is similar to the general mechanism of aging. Oxidative stress caused by ROS, telomere length, and sirtuin activity are considered important factors affecting ovarian aging. The expression of sirtuins (SIRT1, SIRT3, and SIRT6) in the ovaries positively correlates with ovarian reserve [21]. Tamura et al. investigated the protective effect of melatonin on ovarian aging in mice [22]. IVF results showed that the number of fertilized oocytes and the number of blastocysts decreased with aging, but remained at the same level in animals treated with melatonin. Telomere length, which usually decreases with age, and the expression of sirtuin longevity genes (SIRT1, SIRT3) were significantly higher in animals treated with melatonin compared to control mice. MT treatment can activate the levels of SIRT1 and SIRT3 mRNA expression in the ovaries [24].

Thus, there is clear evidence of melatonin, its precursor serotonin, and MT1 and MT2 receptors playing an important role in the functioning of ovaries during the aging process. In our research, we provide a detailed analysis of these signal molecules with the aim of estimating the changing of their expression associated with growing and aging.

We observed a general decrease in MT1 and MT2 expression on the level of mRNA and proteins, assessed by PCR and immunofluorescence staining, respectively. The level decreased dramatically over the first 30 years of life; thereafter, further decrease was less intensive. This corresponds with existing knowledge about the decline in women’s fertility after age 30 [25]. At the gene level, *MTNR1A* and *MTNR1B* expression was extremely depressed in the first 5 years of life. That said, *MTNR1B* expression was significantly higher than *MTNR1A* in all age groups. It seems that MT2 is more involved in neuroendocrine regulation by melatonin in the ovaries than MT1 because of high mRNA expression, but there were no differences between them at the protein level. This can be explained by the different speeds of protein translation and processing, which should be investigated separately.

Additionally, serotonin level, estimated by immunostaining, showed a less intensive decrease during a woman’s aging, reaching a plateau after age 45. It is known that serotonin accumulates in the mammalian ovaries with the involvement of the membrane serotonin transporter SERT and is functionally active in the oocytes of growing follicles, but shows almost no activity in follicular cells [26]. In the mature female rat, analysis for ovarian serotonin content reveals comparatively high serotonin content. The peak for serotonin was observed at estrus. In immature rats, no ovarian serotonin was detected using this procedure [27]. Apparently, the decline in serotonin level observed here can be explained by the start of menopause. The obtained data are crucial to understanding the fundamental mechanisms of aging of the female reproductive system and the search for molecules predicting its aging.

Moreover, studies are emerging on the protective effects of melatonin on the female reproductive system and fertility. Huang et al. (2021) showed that melatonin has the potential to be used as a chemotherapeutic adjuvant to simultaneously improve the outcome of anti-cancer treatment and preserve ovarian function during cisplatin chemotherapy due to its antioxidant properties [28]. Melatonin significantly prevented cisplastin-induced ovarian reserve decline by maintaining Anti-Müllerian hormone (AMH) and bone morphogenetic protein 15 (BMP-15) levels. Melatonin also inhibited cisplastin-induced ovarian inflammation by decreasing IL-1β and IL-18 levels [29]. The addition of melatonin in the medium for oocyte maturation during in vitro fertilization helps to avoid age-related decline in oocyte quality and increase fertility in older reproductive-aged women [30]. When adding it to adipose-derived stem cells (ADSCs), which are potential candidates for the treatment of premature ovarian insufficiency (POI), melatonin restored hormone levels of ADSCs, mean primordial follicle counts, and reproductive capacity in POI mice [31]. At the same time, higher melatonin in follicular fluid and MT2 expression in granulosa cells contribute to the occurrence of ovarian hyperstimulation syndrome [32]. All if the above point to the promising potential of melatonin as a therapeutic agent for enhancing fertility, and of MT1 and MT2 receptors as marker proteins for reproductive age.

Our investigation has the following limitations: We had access to ovarian material from a wide age group of premenopausal and menopausal women—from ages 45 to 59. In the future, it might be interesting to analyze certain age subgroups of premenopausal and menopausal women. We investigated the expression in the ovaries of melatonin receptors and serotonin solely with respect to ovarian aging. Moving forward, it might be important to investigate other markers of neuro-immunoendocrine system aging. Moreover, it should be noticed that despite the authors’ desire to avoid the influence of external factors in this study, not all of them were under our control. As was mentioned above, melatonin synthesis is directly connected with the sleep/wake cycle [33]. Here, we did not avoid this influence, and this could indirectly influence the results of the study.

## 5. Conclusions

The study found that the expression of melatonin receptors 1 and 2 in the ovaries gradually declines from 1 year of age to 90 years. Serotonin expression also declines throughout life, showing a slight plateau in the period from 5 to 18 years of age. The data obtained indicate the potential of MT1 and MT2 melatonin receptors, as well as serotonin, in the ovaries as marker molecules of reproductive age.

## Figures and Tables

**Table 1:** Primer sequences for MTNR1A and MTNR1B genes.

Gene	Sequence
*MTNR1A*	Forward 5′-TGGTTTTGTTGGGTGAAAAG-3′
	Reverse 5′-CTACCCTTACCCTACATAATCCCTATAC-3′
*MTNR1B*	Forward 5′-AGTTGGGTAGGGAAGAGA-3′
	Reverse 5′-AACCCCATACCAACACCCAACAT-3′

**Table 2:** Expression area (%) of MT1, MT2, and serotonin in ovarian tissue of women in different age groups.

Age, y.o.	Me (Q1; Q3)		
	MT1	MT2	Serotonin
Under 12 months	44.59 (38.89; 46.61)	43.66 (43.05; 44.39)	17.36 (16.13; 18.27)
1	30.01 (29.26; 31.41)	26.72 (26.26; 27.46)	30.03 (27.91;30.41)
2–4	19.50 (18.00; 20.16)	15.71 (15.06; 16.11)	15.58 (14.90; 16.71)
5–11	14.86 (14.28; 15.38)	10.47 (9.40; 10.91)	14.31 (13.40;14.89)
12–15	10.71 (10.25; 11.39)	6.12 (5.99; 6.92)	17.28 (16.88; 17.79)
16–18	7.51 (6.98; 7.94)	3.72 (3.38; 4.29)	18.14 (17.67; 18.40)
19–29	3.36 (2.42; 4;56)	2.41 (2.21; 2.50)	11.75 (11.01;12.83)
30–44	4.36 (3.62; 5.84)	2.25 (1.97; 2.56)	6.35 (5.77; 6.55)
45–59	3.32 (2.92; 4.25)	1.44 (1.34; 1.54)	3.07 (2.41; 3.34)
60–74	2.56 (2.41; 3.21)	1.16 (1.03; 1.19)	1.57 (1.34; 2.14)
75–89	1.69 (1.45; 1.78)	0.54 (0.49; 0.65)	0.69 (0.62; 0.87)
>90	0.76 (0.36; 1.05)	0.25 (0.11; 0.46)	0.26 (0.17; 0.29)

**Table 3:** Optical brightness (c.u.) of MT1, MT2, and serotonin in ovarian tissue of women in different age groups.

Age, y.o.	Me (Q1; Q3)		
	MT1	MT2	Serotonin
under 12 months	29.77 (29.04; 31.00)	47.57 (44.68; 48.60)	27.07 (24.05; 29.87)
1	15.60 (14.90; 16.94)	35.97 (34.24; 29.31)	25.45 (23.16; 26.89)
2–4	10.30 (9.11; 10.94)	23.02 (20.09; 24.63)	42.24 (38.94; 45.21)
5–11	7.90 (7.11; 8.06)	14.40 (13.59; 14.86)	21.35 (19.77; 24.80)
12–15	5.82 (5.73; 6.33)	12.07 (11.63; 13.93)	28.77 (24.18; 31.03)
16–18	5.06 (4.86; 5.40)	10.54 (9.76; 11.76)	41.08 (39.61; 42.89)
19–29	3.84 (3.37; 4.14)	5.9 (5.52; 6.83)	21.04 (16.79; 23.66)
30–44	2.82 (2.41; 3.36)	3.89 (3.30; 4.30)	12.47 (11.19; 13.44)
45–59	1.84 (1.59; 2.18)	2.67 (2.51; 2.83)	7.21 (6.64; 8.03)
60–74	0.98 (0.92; 1.05)	2.00 (1.78; 2.78)	4.88 (4.42; 5.42)
75–89	0.61 (0.52; 0.68)	1.80 (1.39; 2.30)	3.39 (2.35; 3.60)
>90	0.34 (0.30; 0.37)	0.43 (0.13; 0.63)	0.83 (0.37; 1.57)

## Data Availability

The datasets presented in this article are not available as they contain confidential information about the concerned patients.
